# Development of an item bank and outcome importance survey for the Australian and New Zealand Bariatric Surgery Registry

**DOI:** 10.1186/s41687-025-00918-w

**Published:** 2025-07-08

**Authors:** Alyssa J. Budin, Priya Sumithran, Andrew D. MacCormick, Ian Caterson, Wendy A. Brown

**Affiliations:** 1 Department of Surgery, School of Translational Medicine, Monash University, Melbourne, Australia; 2 Department of Endocrinology and Diabetes, Alfred Health, Melbourne, Australia; 3 Department of Surgery, The University of Auckland, Auckland, New Zealand; 4 Counties Manukau District Health Board, Otahuhu, Auckland, New Zealand; 5 The Boden Initiative, Charles Perkins Centre, The University of Sydney, Camperdown, Australia; 6 Department of Endocrinology, Royal Prince Alfred Hospital, Camperdown, Australia; 7 Alfred Health, The Alfred Centre, Melbourne, Australia

**Keywords:** Obesity, Bariatric surgery, Patient-reported outcomes, Health-related quality of life, Psychosocial well-being

## Abstract

**Purpose:**

The Australian and New Zealand Bariatric Surgery Registry is developing a bariatric-specific patient-reported outcome measure (PROM) to capture patient outcomes. This study aimed to establish an item bank and questionnaire to assess which outcomes are considered the most important by pre- & post-surgical patients and healthcare practitioners.

**Methods:**

Initial qualitative studies were undertaken to provide an in-depth understanding of patients’ lived experiences, and a targeted literature search was conducted to identify appropriate PROMs. Items from identified PROMs were pooled and categorised to form the basis of a questionnaire developed to interrogate bariatric patients’ and healthcare practitioners’ opinions on the importance of the various outcomes.

**Results:**

1,867 items from 76 instruments were extracted and pooled to form the item bank. Items were categorised and refined to generate an Outcome Importance Questionnaire containing 68 items across 10 domains. 313 participants completed the survey, including 48 pre-surgical patients, 180 post-surgical patients, and 85 Healthcare Practitioners. 52 outcomes (of 68; 76.5%) were prioritised by at least 1 group with ‘Overall mental health’, ‘Co-morbidities’, ‘Satisfaction with surgery’ and ‘Satisfaction with quality of life’ rated as the most important outcomes.

**Conclusion:**

The item bank and outcome importance questionnaire demonstrated good coverage of patient-reported outcomes considered important to all stakeholders. Initial results identified distinct differences in preference votes by patient and healthcare practitioner groups, with sufficient variation to identify those outcomes considered the most important. Additional rounds of testing, including participant-suggested outcomes and forced-choice questions, will facilitate consensus on the most important outcomes for future inclusion in a Registry-based bariatric-specific PROM.

**Supplementary Information:**

The online version contains supplementary material available at10.1186/s41687-025-00918-w.

## Introduction

Bariatric (metabolic) surgery is well-established as the most effective treatment currently available for obesity and its associated medical conditions. The rapidly increasing uptake of these procedures has generated a need for improved assessment and reporting of outcomes in this patient population, particularly in the emerging field of patient-reported outcomes [1, 2]. Recent reviews have identified at least 68 validated measures used in the field, including over 1,000 individual outcomes [1–3].

Research on the impacts of bariatric procedures has been hampered by this heterogeneity of reporting alongside the lack of valid, bariatric-specific patient-reported measures (PRMs) [1, 4, 5]. Due to this, PRMs are especially underutilised in the bariatric field. A study of Australian and Aotearoa New Zealand bariatric surgeons found that 61% of participants reported no collection of any patient-reported measure despite a consensus that such data would be useful [4]. In the United States and England, studies have demonstrated that primary care physicians, general practitioners, and surgeons operating in various subspecialties find PRM data to be useful and impactful on clinical care, provided the PRM was specific to the condition or treatment being assessed and generated actionable data [6–10].

The Australian and New Zealand Bariatric Surgery Registry (The Registry) is currently developing a PRM to incorporate patient outcomes in its data collection, thereby enriching the quality of Registry data and facilitating important ongoing research. As such, the current study aimed to establish an item bank of currently used patient-reported outcomes and to develop and test a questionnaire assessing which outcomes are considered the most important by patient and healthcare practitioner stakeholders. The outcomes deemed most important will be used to select items from the item bank for future testing, psychometric evaluation, and inclusion in a Registry-based bariatric-specific PRM. This will ensure that the eventuating outcomes and measures used are meaningful and beneficial to all end users [2, 4, 11, 12].

## Methods

### Item bank & questionnaire development

Unstructured qualitative and patient-engagement activities conducted by the Registry identified key issues for bariatric surgery patients and their experiences of surgery. This prompted the Registry to investigate the incorporation of a PROM into their regular reporting. An informal literature review was conducted to identify Patient-Reported Outcomes Measures (PROMs) used in people living with obesity, people living with obesity undergoing bariatric surgery, and those awaiting bariatric surgery. The review was guided by a flexible and iterative approach rather than a rigid protocol, allowing for the inclusion of a broad range of sources. Relevant literature was identified through keyword-based searches in academic databases (e.g. PubMed, Google Scholar), the University library and general web searches. Search terms included combinations of: “patient-reported outcome measure,” “PROM,” “assessment tool,” ‘measure,” “questionnaire”, “obesity,” “bariatric surgery,” “preoperative,” “postoperative,” “quality of life,” and “psychosocial outcomes.” Additional sources were identified through manual searches of the reference lists of identified articles and reviews. All relevant PROMs were listed, and the full questionnaire was extracted or obtained through additional searches or requests.

Items from identified PROMs were categorised and pooled by the domains and outcomes assessed. This formed the basis of a questionnaire developed to interrogate bariatric patients’ and healthcare practitioners’ opinions on the importance of the various outcomes. Where possible, similar concepts were combined to facilitate a shorter, user-friendly survey.

### Outcome importance questionnaire testing

The Outcome Importance Questionnaire was used to survey pre- and post-surgical bariatric patients, and a range of healthcare practitioners involved in the treatment and management of bariatric patients, including surgeons, physicians, nurses, dieticians, psychologists, and researchers. The current survey was conducted in Australia only; however, future studies will expand this research to include healthcare practitioners and patients from Aotearoa New Zealand. A copy of the patient version of the Outcome Importance Questionnaire is available in Supplementary Material 2.

Surgeons and healthcare practitioners were recruited via the Australian and New Zealand Bariatric Surgery Registry and the Australian and New Zealand Metabolic & Obesity Surgery Society (ANZMOSS). Pre-surgical patients were recruited via participating healthcare practitioners, and post-surgical patients were randomly selected from the Registry to cover a range of demographics, including age, sex, jurisdiction, public/private operations, diabetes status & type, surgery type, number of revisions, and time since surgery.

Participants completed the survey online via a secure online platform (Qualtrics, Provo, UT), or in paper form. Participants were asked to rate each outcome on a scale from 0 (not important) to 10 (extremely important) by considering the impact each outcome has on a bariatric patient during their surgery and recovery, and how important it would be for doctors and researchers to capture that outcome. Full ethical approval for the study was obtained from the Alfred Hospital Ethics Committee (Project Number: 55/20).

### Analysis of questionnaire responses

Qualitative data were analysed thematically to identify any additional items not originally included in the questionnaire and for feedback on any items that may be inappropriate or unclear. Healthcare practitioners were further sub-categorised as either a medical practitioner (physicians, surgeons), or an allied health practitioner (nurses, dieticians, psychologists, researchers). Responses were organised and analysed by group and subgroup. An outcome was identified as highly important (or prioritised) if at least 70% of participants in any group rated it an 8 or higher. As there are no standardised methods for the analysis of Delphi surveys, these criteria were modelled on similar studies within the field [12–14]. The number and percentage of prioritised outcomes were calculated within and across groups. This was calculated for each individual item in the questionnaire as well as for each domain. Outcomes were then ranked based on the percentage of participants in each group who rated the outcome ≥ 8.

The mean importance score was calculated for each domain by combining the scores for each outcome within a domain (Table 1). Kruskal-Wallis tests and post hoc multiple comparisons with Bonferroni corrections were used to assess differences between groups for each questionnaire item. Differences in demographic variables were assessed using the Kruskal-Wallis test for categorical variables, and Pearson correlations for continuous variables.

**Table 1:** The domains and outcomes used in the development of the item bank and outome importance questionnaire, and the number of proms containing those outcomes

Questionnaire domains	Item bank domains	Outcomes	Number of PROMs
General Health	General Health	General Health / Health Perception	8
		Senses (Vision, Hearing, Breathing, Speech)	3
		Somatic Symptoms	6
		Co-morbidities	1
	Physical Health & Mobility	Physical Health & Exercise	6
		Mobility / Physical Ability	16
	Usual Activities & Self-Care	Usual Activities	10
		Self-Care	1
	Pain		10
	Energy & Fatigue		8
Eating Symptoms	Eating & Digestion	Eating	1
		Appetite	1
		Digestion	3
Sleep	Sleep		4
Sex	Sexual Activity		4
Perception of Surgery	Perception of Surgery	Satisfaction with Surgery	1
Quality of Life	Quality of Life	General Quality of Life / Life Satisfaction	6
		Living	5
		Environment	1
		Achievement	1
		Safety	1
		Future	1
Social Activity	Social	Social Health / Social functioning	10
		Social Isolation	2
		Community	1
		Relationships / Support	10
Mental Health & Emotional Well-Being	Mental Health	General Mental Health	17
		Enthusiasm / Pleasure	1
	Depression & Self-Harm	Depression	15
		Anhedonia	2
		Guilt	1
		Self-Harm / Suicidal Ideation	1
	Anxiety & Control	General Anxiety / Distress	14
		Control	1
		Psychosis	3
		Obsessive-Compulsive	2
		Paranoia	1
		Phobia	3
	Impulsivity & Irritability	Impulsiveness	3
		Addictive Behaviours	1
		Irritability / Frustration / Anger	2
	Cognitive Function	Cognitive Function	3
	Self-Efficacy	Self-Efficacy / Self-Control	2
		Coping	3
Eating Behaviour & Relationship to Food	Eating Behaviour & Relationship to Food		9
Self-Esteem & Body Image	Self-Esteem & Body Image	Confidence / Self-Esteem	5
		General Body Image	11
		Body Image – Weight Preoccupations	5

Statistical analysis was performed using IBM SPSS Statistics (Version 27), with statistical significance inferred at a *p* value of < 0.05.

## Results

As a result of the qualitative review and literature search, 76 instruments were identified (26 generic, 28 disease-specific, and 22 domain/condition-specific), containing 1,867 individual items which were extracted and pooled to form an item bank (Supplementary Material 1, Table S1). Items were categorised according to domain and outcome being measured, generating 19 domains of interest (Table 1).

These domains were further refined to generate the Outcome Importance Questionnaire containing 68 items across 10 domains; (1) General Health; (2) Eating Symptoms; (3) Sleep; (4) Sex; (5) Perception of Surgery; (6) Quality of Life; (7) Social Activity; (8) Mental Health & Emotional Well-Being; (9) Eating Behaviour & Relationship to Food; and (10) Self-Esteem & Body Image (Table 1).

### Outcome importance survey

A total of 313 participants took part in the survey, including 48 pre-surgical patients, 180 post-surgical patients, and 85 Healthcare Practitioners. The characteristics of the participants are presented in Table 2. Pre-surgical patients were younger than post-surgical patients (*p* < 0.001) and there was a higher proportion of women in the patient groups compared to the healthcare practitioner group (*p* < 0.05). The demographics of the patient sample are generally representative of the Australian Registry population [15]. Participants are predominantly female (83.8% vs. 79.7% in the Registry), having most commonly undergone a sleeve gastrectomy (60.0% vs. 67.0% in the Registry), with 28.3% having a revision procedure (compared to 23.6% in the Registry).

**Table 2:** Characteristics of pre-surgical patients, post-surgical patients, and healthcare practitioners participating in the study (*n* = 313)

Patients (*n* = 228)		
	**Pre-Surgical** ***(n* = 48)**	**Post-Surgical** ***(n* = 180)**
**Mean Age (SD)**	42.3 (9.8)	49.2 (12.3)
**Number Female (%)**	43 (89.6)	148 (82.2)
**Ethnicity (%)**		
Non-Indigenous Australian	38 (79.2)	141 (78.3)
Indigenous Australian or Torres Strait Islander	2 (4.2)	9 (78.3)
European	3 (6.3)	10 (5.6)
Other	5 (10.4)	20 (11.1)
**Employment (%)**		
Working full-time / Self-Employed	26 (54.2)	102 (56.7)
Working part-time / Casual	13 (27.1)	36 (20.0)
Retired	-	13 (7.2)
Unable to work	3 (6.3)	5 (2.8)
Home Duties	1 (2.1)	10 (5.6)
Student / Apprentice	1 (2.1)	6 (3.3)
Unemployed	1 (2.1)	3 (1.7)
Others	3 (6.3)	5 (2.8)
**Education (%)**		
Less than Year 12 or equivalent	9 (18.8)	32(17.8)
Year 12 or equivalent	11 (22.9)	33 (18.3)
Trade / Technical / Vocational qualification	11 (22.9)	56 (31.1)
Undergraduate degree	11 (22.9)	30 (16.7)
Postgraduate degree	6 (12.5)	28 (15.6)
**Bariatric Procedure (%)**^(a)^		
Adjustable Gastric Band (AGB)	-	39 (21.7)
Sleeve Gastrectomy (SG)	41 (85.4)	108 (60.0)
Roux-en-Y Gastric Bypass (RYGB)	3 (6.3)	22 (12.2)
One Anastomosis Gastric Bypass (OAGB)	2 (4.2)	7 (3.9)
Other	-	4 (2.2)
Not Sure	2 (4.2)	-
**Mean time since surgery (SD)**	-	39.9 (53.4) months Range: 0–238
**Healthcare Practitioners** (***n*** = 85)		
**Number Female (%)**	48 (56.5)	
* Prefer not to say*	1 (1.2)	
**Profession (%)**		
Bariatric Surgeon	36 (42.4)	
Dietician	22 (25.8)	
Nurse Specialist / Nurse Practitioner	15 (17.7)	
Psychologist / Psychiatrist	7 (8.3)	
Bariatric Physician / GP	3 (3.5)	
Researcher	2 (2.4)	
**Years in Profession (%)**		
1–5 years	25 (29.4)	
6–10 years	18 (21.2)	
More than 10 years	42 (49.4)	
**Ethnicity (%)**		
Non-Indigenous Australian	58 (68.2)	
Indigenous Australian or Torres Strait Islander	-	
European	11 (12.9)	
Other	14 (16.5)	
*Prefer not to say*	2 (2.4)	

SD standard deviation

^(a)^ Planned procedure for pre-surgical patients; primary procedure for post-surgical patients

### Patient-reported outcome importance

Items rated 8–10 by 70% of participants (highly important) by any group are presented in Table 3. All items prioritised by any group or subgroup are presented in Supplementary Material 1, Table S2. The highest-rated items were ‘Overall Mental Health’ for pre-surgical patients (93.8% rated ≥ 8), ‘Satisfaction with Quality of Life’ for post-surgical patients (86.1% rated ≥ 8), and ‘Co-morbidities’ for healthcare practitioners (92.9% rated ≥ 8).

**Table 3:** Items rated highly important (≥ 70% rating the item ≥ 8) by any group (Pre-surgical patients, Post-Surgical patients, and healthcare Practitioners)

Item	Pre-surgical patients	Post-surgical patients	Healthcare practitioners	Sig.
	(*n* = 48)	(*n* = 180)	(*n* = 85)	
	% ≥ 8	% ≥ 8	% ≥ 8	
**Items rated highly important by all groups**				
Overall mental health	93.80%	75.00%	70.60%	**0.004**
Co-morbidities * E.g. diabetes*,* hypertension*,* sleep apnoea*	81.30%	77.20%	92.90%	**0.009**
Satisfaction with surgery	91.70%	78.90%	72.90%	**0.001**
Satisfaction with quality of life	91.70%	86.10%	80.00%	**0.020**
**Items rated highly important by pre- and post-surgical patients**				
Normality * (feeling able to live a “normal” life)*	87.50%	78.90%	61.20%	**0.006**
Self-esteem / Self-confidence	85.40%	70.00%	62.40%	**0.005**
Feeling in control of weight and appearance	83.30%	75.60%	50.60%	**< 0.001**
Energy Levels / Fatigue	81.30%	70.00%	65.90%	0.059
Outlook on life and expectations for the future	79.20%	74.40%	50.60%	**< 0.001**
Eating patterns * (healthy and balanced eating patterns)*	77.10%	71.70%	63.50%	0.221
**Items rated highly important by pre-surgical patients and healthcare practitioners**				
Decision remorse * (feeling of anxiety or regret about the decision to undergo surgery)*	83.30%	67.20%	81.20%	**< 0.001**
Mobility * E.g. ability to walk*,* climb stairs*,* lift/carry groceries*,* bend or kneel*	79.20%	67.20%	75.30%	0.177
General physical health * E.g. fitness*,* strength*,* endurance*	75.00%	65.60%	78.80%	0.130
**Items rated highly important by pre-surgical patients only**				
Level of social activity	83.3%	69.4%	63.5%	**0.019**
Depression	83.3%	63.9%	63.5%	**0.014**
Weight / Surgery-specific symptoms * E.g. vomiting*,* regurgitation*,* heartburn*,* nausea*,* shortness of breath*	81.3%	63.9%	64.7%	**0.046**
Ability to eat different types of food	77.1%	53.9%	64.7%	**0.019**
Preoccupation with thoughts about body shape and/or size	77.1%	62.2%	56.5%	**0.005**
Relationship with spouse/partner or developing intimate relationships	77.1%	64.4%	55.3%	**0.038**
Anxiety	77.1%	55.0%	57.6%	**0.005**
Coping * (ability to deal with stress or difficulties)*	77.1%	51.7%	48.2%	**0.003**
Ability to care for oneself * E.g. dressing*,* bathing*,* grooming*,* or eating*	77.1%	68.3%	68.2%	**0.048**
Suicidal thoughts	75.0%	48.3%	64.7%	**0.001**
Snoring * (which wakes the snorer or affects others)*	75.0%	62.2%	56.5%	**0.023**
Feeling in control of thoughts and feelings	75.0%	56.1%	44.7%	**0.002**
Confidence to engage in social activity	75.0%	67.2%	63.5%	**0.043**
Level of pain	72.9%	55.6%	58.8%	0.091
Mood swings	72.9%	56.7%	41.2%	**0.004**
Self-harm behaviours or thoughts	72.9%	48.9%	61.2%	**0.004**
Self-efficacy * (belief in own ability to succeed)*	72.9%	57.8%	48.2%	0.061
Binge eating	70.8%	60.6%	64.7%	0.096
Cognitive function * E.g. concentrating*,* problem-solving*,* remembering*	70.8%	62.8%	48.2%	**0.042**
Preoccupation with thoughts of food	70.8%	58.9%	61.2%	0.103
Overall quality of life, health and well-being	70.8%	64.4%	54.1%	**0.042**
Emotional eating	70.8%	62.2%	63.5%	0.224
**Items rated highly important by healthcare practitioners only**				
Medication use	68.8%	67.2%	78.8%	0.570

Items are presented as they appeared in the questionnaire. Results are presented as percentage of participants within each group ranking the item ≥ 8

Significance indicates differences between groups; pre-surgical patients, post-surgical patients and healthcare practitioners

Pre-surgical patients, post-surgical patients, and healthcare practitioners rated 35, 10 and 8 items as highly important, respectively. There were 4 overlapping items (out of 68, 5.9%) common to all three groups, 6 overlapping items (8.8%) between the two patient groups, and 3 (4.4%) between pre-surgical patients and healthcare practitioners. Unanimously prioritised outcomes included ‘Overall mental health’, ‘Co-morbidities’, ‘Satisfaction with surgery’ and ‘Satisfaction with quality of life’ (Table 3). There were 23 items (3.8%) prioritised by a single group with significant differences between the groups for 36 items (52.9%). Examples of discordant items prioritised by pre-surgical patients included ‘Level of social activity’, ‘Relationship with spouse/partner’, ‘Depression’, ‘Anxiety’, ‘Suicidal thoughts’, Binge eating’, and ‘Emotional eating’ while ‘Medication Use’ was the only discordant outcome prioritised by Healthcare Practitioners (Table 3).

Pre-surgical patients prioritised 9 physical outcomes (of 25; 36.0%) and 26 psychosocial outcomes (of 43; 60.5%), post-surgical patients prioritised 2 physical outcomes (8.7%) and 8 psychosocial outcomes (18.6%) and healthcare practitioners prioritised 4 physical outcomes (16.0%) and 4 psychosocial outcomes (9.3%).

The percentage of Medical and Allied Health Practitioner sub-groups prioritising items is presented in Supplementary Material 1, Table S2. Of the 8 items prioritised by healthcare practitioners, there were 5 overlapping items between the subgroups, and 3 items prioritised by Allied Health Practitioners only. There were an additional 35 items prioritsied by Allied Health Practitioners, 16 of which were not prioritised by any other group, including ‘Addictive behaviours’, ‘Pain interference with day-to-day activities’, ‘Change in appetite’, ‘Satisfaction with sleep’, and ‘Experience of stigma or discrimination’ (Supplementary Maerial 1, Table S2). Medical practitioners prioritised 2 physical outcomes (8.7%) and 3 psychosocial outcomes (7.0%) while Allied Health Practitioners prioritised 17 physical outcomes (68.0%) and 26 psychosocial outcomes (60.5%).

Mean combined scores for the overall domains of interest for each group are presented in Table 4. The domain with the highest mean rating was ‘Perception of Surgery’ for pre-surgical patients (9.33 ± 0.83) and healthcare practitioners (8.84 ± 1.07) and ‘Quality of Life’ for post-surgical patients (8.42 ± 1.45). There was a significant difference in mean scores between groups on 6 of the 10 domains.

**Table 4:** Average importance scores and rank for pre-surgical patients, post-surgical patients, and healthcare practitioners for each domain.

Importance rating							
Domain	Pre-Surgical patients		Post-Surgical patients		Healthcare practitioners		Sig.
	(*n* = 48)		(*n* = 180)		(*n* = 85)		
	Mean (SD)	Rank	Mean (SD)	Rank	Mean (SD)	Rank	
Perception of Surgery	9.33 (0.83)	1	8.17 (1.77)	2	8.84 (1.07)	1	**< 0.001**
Quality of Life	8.86 (1.38)	2	8.42 (1.45)	1	8.11 (1.39)	3	**0.018**
Self-Esteem & Body Image	8.62 (1.50)	3	7.96 (1.65)	3	7.78 (1.87)	5	**0.024**
General Health	8.33 (1.32)	4	7.78 (1.51)	4	8.08 (1.24)	4	**0.04**
Social Well-Being	8.24 (1.49)	5	7.59 (1.76)	5	7.74 (1.69)	6	0.07
Mental Health & Emotional Well-Being	8.24 (1.51)	6	7.32 (2.03)	8	7.55 (1.75)	7	**0.014**
Eating Behaviour & Relationship to Food	8.10 (2.05)	7	7.55 (2.29)	6	8.22 (1.68)	2	**0.043**
Sleep	8.05 (1.87)	8	7.49 (1.85)	7	7.48 (1.76)	8	0.144
Sex	7.45 (2.62)	9	7.01 (2.42)	9	6.76 (2.20)	10	0.288
Eating Symptoms	6.79 (1.86)	10	6.92 (2.05)	10	7.05 (1.85)	9	0.759

Mean scores for the overall domains for Healthcare Practitioner subgroups identified the highest-rated domains for Medical Practitioners to be ‘Perception of Surgery’ (8.35 ± 1.14), ‘Quality of Life’ (7.47 ± 1.46) and ‘General Health’ (7.45 ± 1.28) and for Allied Health Practitioners; ‘Perception of Surgery’ (9.30 ± 0.78), ‘Eating Behaviours & Relationship to Food’ (9.17 ± 1.01) and ‘Self-Esteem & Body Image’ (8.76 ± 1.05). There were significant differences between the subgroups in all domains except for ‘Sex’.

There were 22 additional items raised by participants completing the survey. This included 7 physical outcomes; ‘Development of excess skin’, ‘Fertility’, ‘Hormonal changes’, ‘Ability to tolerate fluids’, ‘Variation in food/fluid tolerance’, and ‘Maladaptive eating habits’, and 15 psychosocial outcomes; ‘Comfort in social eating situations’, ‘Impact of physical outcomes on social life’, ‘Feeling in control of eating behaviour’, ‘Impact of mental health on eating behaviour’, ‘Feeling conscious of surgical outcomes’, ‘Fear of surgical outcomes affecting others’, ‘Body dysmorphia’, ‘Desire for ‘cosmetic’ procedures’, ‘Unmet expectations’, ‘Feelings of Guilt’, ‘Comparison to others’, ‘Fear of weight regain’, ‘Fear of returning habits’, ‘Preparedness for surgery’, and ‘Surgery secrecy’.

## Discussion

This study included multiple qualitative methods and targeted searches in the development of a large item bank including 1,867 items from existing patient-reported outcome measures (PROMs). Categorisation of this item bank facilitated the generation of an outcome importance questionnaire that included 68 outcomes across 10 domains of interest. The development of the item bank and questionnaire using these methods allowed for the inclusion of items and outcomes currently considered important to collect in the bariatric field. The variety of outcomes included in the questionnaire exceeds those usually found in existing bariatric-specific and generic PROMs which tend to focus on physical outcomes such as weight loss, diabetes resolution, changes in mobility, and digestive symptoms as well as general health-related quality of life. By including a wider range of outcomes in our outcome importance questionnaire, we have a better chance of capturing those outcomes that patients and healthcare providers consider the most important and impactful.

Initial testing of the outcome importance questionnaire facilitated important insights into the contrasts between patient and healthcare practitioner opinions, including 39 items prioritised by only a single group or sub-group. The differences between patient and healthcare practitioner importance valuations have been widely investigated. In general, the literature suggests that patients tend to rate psychosocial outcomes such as quality of life, mental health, and emotional well-being higher than healthcare practitioners, while practitioners tend to favour clinical outcomes such as the resolution of comorbidities and physical functioning [12, 14, 16–20]. This observation was maintained in our study with pre- and post-surgical patients prioritising a higher percentage of psychosocial outcomes (36.0% and 18.6%, respectively) compared to healthcare practitioners (9.3%). In addition, allied health practitioners prioritised a much higher percentage of psychosocial outcomes (60.5%) compared to medical practitioners (7.0%), consistent with current literature [14].

Although these results highlight a clear and substantial discordance in the number and type of outcomes prioritised between the groups, there was a good level of agreement on the mean importance scores for each domain. ‘Perception of Surgery’ and ‘Quality of Life’ were in the top 3 for all groups while ‘Sex’ and ‘Eating Symptoms’ were the bottom two. The most significant difference was for the domain ‘Eating Behaviour and Relationship to Food’ which healthcare practitioners ranked significantly higher than the two patient groups.

There was a clear ceiling effect noted in the pre-surgical group with multiple outcomes having a median score of 10 (out of 10). These results highlight the need for additional rounds of testing, utilising Delphi methods to approach consensus on which outcomes are the most important. Ranking questions will also be included moving forward to engage participants in a forced choice paradigm so that we may better understand how patients and healthcare practitioners value the various outcomes. There were 22 additional outcomes suggested by participants during the questionnaire testing. These outcomes were not identified in the initial qualitative work and are not currently included in the existing PROMs used to generate the item bank and questionnaire. This further highlights the gaps in the currently utilised PROMs in the field and signifies the importance of collecting stakeholder input to ensure all important outcomes are identified.

Although the sample is generally representative, results are still limited to a predominantly non-Indigenous Australian cohort. Future work will involve the expansion of this work in Aotearoa New Zealand to compare and validate our work in this population. There will also be a focus on ethnic minority groups, particularly indigenous Aboriginal and Torres Strait Islander peoples in Australia and Māori in Aotearoa New Zealand, to better understand how outcomes may differ in these patient populations. In addition, this work may be relevant to other cohorts of people living with obesity who are undergoing non-surgical obesity treatment, and this is an avenue the Registry will consider moving forward.

Additional rounds of testing using the Outcome Importance Questionnaire, including the additional items proposed by participants, will be conducted to establish consensus using Delphi methods. This will facilitate the selection of items from our item bank for the development of a bariatric-specific PROM for the Registry. Future research will involve continued pilot testing and validation of the selected items using modern psychometric techniques and additional qualitative work with patients and healthcare practitioners to ensure the resulting PROM is meaningful, valid, and useful to all end users.

## Conclusion

Data from this study has been used to establish an item bank of currently used patient-reported outcomes and to develop and test a questionnaire assessing which of these outcomes are considered the most important by patient and healthcare practitioner stakeholders. Initial deployment of the outcome importance questionnaire identified distinct differences in preference votes by patient and healthcare practitioner groups, with sufficient variation to identify those outcomes considered the most important. Additional rounds of testing, including participant-suggested outcomes and forced-choice questions will facilitate consensus on the most important outcomes. These outcomes will be used to select items from the item bank for future testing, psychometric evaluation and inclusion in a Registry-based bariatric-specific patient-reported measure.

## Electronic supplementary material

Below is the link to the electronic supplementary material.

Supplementary Material 1

Supplementary Material 2

## Data Availability

The datasets used and/or analysed during the current study are available from the corresponding author on reasonable request.
