# Supplementary material for: Development of an item bank and outcome importance survey for the Australian and New Zealand Bariatric Surgery Registry
Source: J Patient Rep Outcomes. 2025 Jul 8;9:85. doi: 10.1186/s41687-025-00918-w (PMC12238453; doi:10.1186/s41687-025-00918-w)
Supplement: Supplementary file 1 — Supplementary Material 1 [file 41687_2025_918_MOESM1_ESM.pdf]

Journal of Patient-Reported Outcomes

Development of an Item Bank and Outcome Importance Survey for the Australian and New Zealand Bariatric Surgery Registry

Supplementary Material

Table S1. Validated PROMs identified in the literature search.

Table S2. Items rated highly important (≥ 70% rating the item ≥ 8) in Round One by Pre-surgical Patients, Post-Surgical Patients, and Healthcare Practitioners, including Medical and Allied Health Practitioner subgroups.

Table S1. Validated PROMs identified in the literature search

PROM	Domains	
**Generic measures**		
15D	Mobility<br>Vision<br>Hearing<br>Breathing<br>Sleeping<br>Eating<br>Speech<br>Excretion	Usual activities<br>Mental function<br>Discomfort and symptoms<br>Depression<br>Distress<br>Vitality<br>Sexual activity
AQoL 8	Independent living<br>Relationships<br>Senses<br>Mental Health	
AQoL-4D	Independent living<br>Relationships<br>Senses<br>Mental Health	
AQoL-6D	Independent living<br>Relationships<br>Senses	Mental Health<br>Coping<br>Pain
AQoL-8D	**Physical**<br>Independent living<br>Senses<br>Pain	**Psychosocial**<br>Mental health<br>Happiness<br>Self-worth<br>Coping<br>Relationships
BASIS-32	Relation to self and others<br>Daily Living & Role Functioning<br>Depression & Anxiety	Impulsive & Addictive behaviour<br>Psychosis
EQ-5D-5L	Mobility<br>Self Care<br>Usual Activites	Pain/Discomfort<br>Anxiety/Depression
GWB<br>*General Well-being Scale*	Anxiety<br>Depression<br>General health	Positive well-being<br>Self-control<br>Vitality
HALex<br>*Health and Activity Limitation Index*	Percieved health<br>Activity limitation	
M-A QoLQII	Self-esteem<br>Physical well-being<br>Social relationships	Work<br>Sexuality<br>Eating behaviour
MOS-SS<br>*Medical Outcomes Study Sleep Scale*	Sleep initiation<br>Maintenance<br>Respiratory problems	Quantity<br>Perceived adequacy<br>Somnolence
MOS-SSS<br>*Medical Outcomes Study Social Support Survey*	Emotional/informational support<br>Tangible support<br>Affectionate support<br>Positive social interaction	
MSPSS<br>*Multidimensional Scale of Perceived Social Support*	Social support from:<br>Family<br>Friends<br>Significant other	
NHP<br>*Nottingham health profile*	Energy level<br>Pain<br>Emotional reaction<br>Sleep	Social isolation<br>Physical abilities<br>Life areas affected
PQoL<br>*Perceived Quality of Life*	Physical<br>Social<br>Cognitive	
PROMIS-10<br>*Patient-Reported Outome Measurement Information System*	Physical health<br>Mental health<br>Social health	Pain<br>Fatigue<br>Perceived QoL
PROMIS-29	Physical function<br>Anxiety<br>Depression<br>Fatigue	Sleep disturbance<br>Ability to participate in social roles and activities<br>Pain interference/intensity

PWI<br>*Personal Wellbeing Index*	Standard of living<br>Health<br>Achieving in life<br>Relationships	Safety<br>Community- connectedness<br>Future security
QWB-SA<br>*Quality of well-being scale, self-administered*	Health symptoms/aides<br>Feelings, thoughts & behaviours<br>Physical activity/mobility<br>General health	
SCL90-R<br>*Symptom checklist 90-R*	Anxiety<br>Depression<br>Hostility<br>Interpersonal sensitivity<br>Obsessive-compulsive behaviour	Paranoid ideation<br>Phobic anxiety<br>Psychoticism<br>Somatic symptoms
SF-12	General health<br>Physical functioning<br>Role limitations physical<br>Role limitations emotional	Pain<br>Emotional well-being<br>Social functioning
SF-20	Physical functioning<br>Role functioning<br>Social functioning	Pain<br>General health perceptions<br>Mental Health
SF-36	Physical functioning<br>Role limitations physical<br>Role limitations emotional<br>Energy/fatigue	Emotional well-being<br>Social functioning<br>Pain<br>General health
SF-6D	Physical functioning<br>Role limitations<br>Energy/fatigue	Emotional well-being<br>Social functioning<br>Pain
SSI<br>*Social Support Inventory*	Emotional support<br>Informative support<br>Social companionship<br>Instrumental support	
WHOQOL-BREF	Physical health<br>Psychological<br>Social relationships<br>Environment	
**Disease-specific measures**		
BAQ<br>*Body Attitudes Questionnaire (Ben-Tovim Walker BAQ)*	Feelings of overall fatness<br>Self-disparagement<br>Strength / Fitness	Salience of weight<br>Feelings of attractiveness<br>Consciousness of lower body fat
BICSI<br>*Body Image Coping Strategies Inventory*	Avoidance<br>Appearance fixing<br>Positive rational acceptance	
BIDQ<br>*Body Image Disturbance Questionnaire*	Body image disturbance	
BISS<br>*Body Image States Scale*	Dis/satisfaction with one's overall physical appearance<br>Dis/satisfaction with one's body size and shape<br>Dis/satisfaction with one's weight<br>Feelings of physical un/attractiveness<br>Current feelings about one's looks relative to how the average person looks<br>Evaluation of one's appearance relative to how the average person looks	
BODY-Q	Appearance<br>HRQoL	
BOSS<br>*Bariatric and Obesity-Specific Survey*	**Generic dimensions**<br>Incapacity<br>Work and well-being<br>Social functioning	**Bariatric-specific domains**<br>Appearance and health<br>Eating patterns<br>Sexual health
BQL<br>*Bariatric Quality of Life Index*	Psychological well-being<br>Social functioning<br>Physical functioning	Problems/symptoms related to obesity surgery<br>Obesity-related co-morbidity
BSQ-8A/B/C/D / BSQ-16A/B / BSQ-32<br>*Body Shape Questionnaire*	Concern with body shape	
BUT<br>*Body Uneasiness Test*	Weight phobia<br>Body image concerns<br>Avoidance	Compulsive self-monitoring<br>Depersonalisation
DEBQ<br>*Dutch Eating Behaviour Questionnaire*	Restrained eating<br>Emotional eating<br>External eating	

EAT-26<br>*Eating Attitudes Test*	Disordered eating behaviour
EDE-Q<br>*Eating Disorder Examination Questionnaire*	Restraint<br>Eating Concern<br>Shape Concern<br>Weight Concern
GIQLI<br>*Gastrointestinal QoL Index*	Physical role<br>Emotional role<br>Large bowel function<br>Upper GI tract function<br>Meteorism
IWQOL-Lite<br>*Impact of Weight on QoL*	Physical function<br>Self-esteem<br>Sexual life<br>Public distress<br>Work
IWQOL-Lite-CT<br>*Impact of Weight on QoL Clinical Trial Version*	Physical<br>Physical Function<br>Psychological
MBSRQ<br>*Multi-dimensional body self-relations questionnaire*	Appearance evaluation<br>Appearance orientation<br>Fitness evaluation<br>Fitness orientation<br>Health evaluation<br>Health orientation<br>Illness orientation<br>Body areas satisfaction<br>Overweight preoccupations<br>Self-classified weight
OP-scale<br>*Obesity-related Problems scale*	Psychosocial functioning and well-being
ORCD<br>*Obesity-Related Coping and Distress Scales*	**Coping**<br>Social trust<br>Fighting spirit<br>Wishful thinking<br>**Distress**<br>Intrusion<br>Helplessness
ORWELL-97<br>*Obesity Related well-being questionnaire*	Psychological status & social adjustment<br>Physical symptoms impairment
OWLQOL<br>*Obesity and Weight-Loss Quality of Life Questionnaire*	Quality of life
PBOT<br>*Post-Bariatric Outcome Tool*	Appearance<br>Physical health
PFS<br>*The Power of Food Scale*	**Appetite for:**<br>Food available<br>Food present<br>Food tasted
QOLOS<br>*Quality of Life for Obesity Surgery*	**Section 1**<br>Eating disturbance<br>Physical functioning<br>Body satisfaction<br>Family support<br>Social discrimination<br>Positive activities<br>Partnership<br>**Section 2**<br>Excess skin<br>Eating adjustment<br>Dumping<br>Satisfaction with surgery
TFEQ-R18<br>*Three-Factor Eating Questionnaire - R18*	Cognitive restraint<br>Uncontrolled eating<br>Emotional eating
TRIM-Weight<br>*Treatment-Related Impact Measure of Weight*	Daily life<br>Weight management<br>Treatment burden<br>Experience of side effects<br>Psychological health
WRSM<br>*Weight-Related Symptoms Measure*	Degree of bothersomeness for each symptom
**Domain/condition specific measures**	
BDI<br>*Beck Depression Inventory*	Depression
BFNE<br>*Brief Fear of Negative Evaluation Scale*	Fear of negative evaluation,<br>Hallmark criteria for the diagnosis of social phobia
BIS-11<br>*Barrat Impulsiveness Scale*	**Attentional**<br>Attention<br>Cognitive instability<br>**Motor**<br>Motor<br>Perseverance<br>**Non-planning**<br>Self-control<br>Cognitive complexity
BIS-15<br>*Barratt Impulsiveness Scale Short-Form*	Attentional impulsivity<br>Motor impulsivity<br>Non-planning impulsivity

CES-D<br>*Center for Epidemiological Studies Depression Scale*	Sadness (dysphoria<br>Loss of interest (anhedonia)<br>Appetite<br>Sleep<br>Thinking/concentration<br>Guilt (worthlessness)<br>Tired (fatigue)<br>Movement (agitation)<br>Suicidal ideation
CSEI<br>*The Coopersmith Self-Esteem Inventory*	Self-esteem
DASS-21	Depression<br>Anxiety<br>Stress
GAD-7<br>*Generalised Anxiety Disorder 7-item Scale*	Anxiety
GSE<br>*General Self-Efficacy Scale*	Self-efficacy
HADS<br>*Hospital Anxiety and Depression Scale*	Anxiety<br>Depression
K10<br>*Kessler Psychological Distress Scale*	Distress (Anxiety & depression)
MHI<br>*Mental Health Inventory (from MOS)*	**Psychological distress**<br>Anxiety<br>Depression<br>Loss of behavioural/emotional control<br>**Psychological well-being**<br>Positive affect<br>Emotional ties<br>Life satisfaction
MHQ<br>*Middlesex Hospital Questionnaire*	Free-floating anxiety<br>Phobic anxiety<br>Obsessive-compulsive traits & symptoms<br>Somatic symptoms<br>Depressive symptoms<br>Hysteria
PHQ-2 / PHQ-9<br>*Patient Health Questionnaire*	Depression
RDS<br>*RAND Depression Screener*	Major depressive and dysthymic disorders
RSES<br>*Rosenberg Self-Esteem Scale*	Self-esteem
UPPS-P / UPPS-P-SF<br>*Urgency, Premeditation (lack of), Perseverance (lack of), Sensation Seeking, Positive Urgency Impulsive Behaviour Scale*	**Emotion-based rash action**<br>Negative urgency<br>Positive urgency<br>**Sensation seeking**<br>**Deficits in conscientiousness**<br>(lack of) premeditation<br>(lack of) perseverance
WEL<br>*The Weight Efficacy Lifestyle Questionnaire*	Negative emotions<br>Availability<br>Social pressure<br>Physical discomfort<br>Positive activities
WEL-SF<br>*The Weight Efficacy Lifestyle Questionnaire Short Form*	Eating self-efficacy
ZSDS<br>*Zung Self-Rating Depression Scale*	The pervasive effect<br>The physiological equivalents<br>Other disturbances<br>Psychomotor activities

Table S2. Items rated highly important (≥ 70% rating the item ≥ 8) in Round One by Pre-surgical Patients, Post-Surgical Patients, and Healthcare Practitioners, including Medical and Allied Health Practitioner subgroups.

Item	Pre-Surgical Patients (n = 48)		Post-Surgical Patients (n = 180)		Healthcare Practitioners (n = 85)		Sig. [(a)]	Medical Practitioners (n = 39)		Allied Health Practitioners* (n = 46)		Sig. [(b)]
	Md (IQR)	% ≥ 8	Md (IQR)	% ≥ 8	Md (IQR)	% ≥ 8		Md (IQR)	% ≥ 8	Md (IQR)	% ≥ 8	
**Items rated highly important by all groups**												
Overall mental health	10 (9 - 10)	93.8%	9 (8 - 10)	75.0%	9 (8 - 10)	70.6%	0.004	8 (6.5 - 9)	56.4%	10 (9 - 10)	82.6%	0.000
Co-morbidities<br>*E.g. diabetes, hypertension, sleep apnoea*	9.5 (8 - 10)	81.3%	9 (8 - 10)	77.2%	10 (9 - 10)	92.9%	0.009	9 (8 - 10)	94.90%	10 (9 - 10)	91.30%	0.004
Satisfaction with surgery	10 (9 - 10)	91.7%	9 (8 - 10)	78.9%	9 (8 - 10)	72.9%	0.001	8 (7.5 - 9.5)	71.8%	9 (8 - 10)	73.9%	0.023
Satisfaction with quality of life	10 (9 - 10)	91.7%	9 (8 - 10)	86.1%	9 (8 - 10)	80.0%	0.020	8 (8 - 9)	74.4%	10 (9 - 10)	84.8%	0.000
**Items rated highly important by pre- and post-surgical patients**												
Normality<br>(feeling able to live a "normal" life)	10 (8 - 10)	87.5%	9 (8 - 10)	78.9%	9 (7 - 10)	61.2%	0.006	7 (6 - 9)	41.0%	9 (8 - 10)	78.3%	0.000
Self-esteem / Self-confidence	10 (8 - 10)	85.4%	8 (7 - 10)	70.0%	8 (7 - 10)	62.4%	0.005	7 (7 - 8.5)	43.6%	9 (8 - 10)	78.3%	0.000
Feeling in control of weight and appearance	10 (8 - 10)	83.3%	8 (8 - 10)	75.6%	8 (6.5 - 9)	50.6%	<0.001	7 (5.5 - 8)	30.8%	9 (8 - 10)	67.4%	0.000
Energy Levels / Fatigue	8.5 (8 - 10)	81.3%	8 (7 - 10)	70.0%	8 (7 - 10)	65.9%	0.059	7 (6 - 8)	38.5%	9 (8 - 10)	89.1%	0.000
Outlook on life and expectations for the future	9 (8 - 10)	79.2%	9 (8 - 10)	74.4%	8 (6.5 - 8.5)	50.6%	<0.001	7 (6 - 8)	43.6%	8 (7 - 9)	56.5%	0.067
Eating patterns<br>*(healthy and balanced eating patterns)*	10 (8 - 10)	77.1%	9 (7 - 10)	71.7%	9 (7 - 10)	63.5%	0.221	7 (6 - 8.5)	43.6%	10 (9 - 10)	80.4%	0.000
**Items rated highly important by pre-surgical patients and healthcare practitioners**												
Decision remorse<br>*(feeling of anxiety or regret about the decision to undergo surgery)*	10 (8.5 - 10)	83.3%	8 (7 - 10)	67.2%	9 (8 - 10)	81.2%	<0.001	8 (8 - 9)	76.9%	10 (9 - 10)	84.8%	0.000
Mobility<br>*E.g. ability to walk, climb stairs, lift/carry groceries, bend or kneel*	9 (8 - 10)	79.2%	8 (7 - 10)	67.2%	8 (7.5 - 9)	75.3%	0.177	8 (7 - 9)	59.0%	9 (8 - 10)	89.1%	0.005
General Physical Health<br>*E.g. fitness, strength, endurance*	8.5 (7.25 - 10)	75.0%	8 (7 - 10)	65.6%	9 (8 - 10)	78.8%	0.130	8 (7 - 9)	66.7%	10 (8 - 10)	89.1%	0.001
**Items rated highly important by pre-surgical patients only**												
Level of social activity	10 (8 - 10)	83.3%	8 (7 - 10)	69.4%	8 (7 - 10)	63.5%	0.019	7 (6 - 9)	46.2%	9 (8 - 10)	78.3%	0.000
Depression	9.5 (8 - 10)	83.3%	8 (7 - 10)	63.9%	8 (7 - 10)	63.5%	0.014	8 (6 - 8)	51.3%	9 (8 - 10)	73.9%	0.000
Weight / Surgery-specific symptoms<br>*E.g. vomiting, regurgitation, heartburn, nausea, shortness of breath*	9 (8 - 10)	81.3%	8 (7 - 10)	63.9%	8 (7 - 9)	64.7%	0.046	8 (6 - 9)	53.8%	8 (7 - 10)	73.9%	0.065
Ability to eat different types of food	8 (8 - 10)	77.1%	8 (6 - 9)	53.9%	8 (7 - 10)	64.7%	0.019	7 (6 - 9)	46.2%	9 (8 - 10)	80.4%	0.000
Preoccupation with thoughts about body shape and/or size	10 (8 - 10)	77.1%	8 (7 - 10)	62.2%	8 (7 - 10)	56.5%	0.005	7 (5.5 - 8)	33.3%	9 (8 - 10)	76.1%	0.000
Relationship with spouse/partner or developing intimate relationships	9 (8 - 10)	77.1%	8 (7 - 10)	64.4%	8 (7 - 10)	55.3%	0.038	7 (5 - 9)	38.5%	9 (8 - 10)	69.6%	0.002
Anxiety	10 (8 - 10)	77.1%	8 (6 - 10)	55.0%	8 (6 - 10)	57.6%	0.005	7 (5 - 8)	41.0%	9 (8 - 10)	71.7%	0.000
Coping<br>*(ability to deal with stress or difficulties)*	9 (8 - 10)	77.1%	8 (6 - 10)	51.7%	8 (6 - 9)	48.2%	0.003	6 (5 - 8)	28.2%	8 (7.25 - 10)	65.2%	0.000
Ability to care for oneself<br>*E.g. dressing, bathing, grooming, or eating*	10 (8 - 10)	77.1%	8 (7 - 10)	68.3%	8 (7 - 10)	68.2%	0.048	8 (6 - 9)	51.3%	9 (8 - 10)	82.6%	0.004
Suicidal thoughts	9.5 (8 - 10)	75.0%	7.5 (4 - 10)	48.3%	9 (7 - 10)	64.7%	0.001	8 (5 - 9)	48.7%	10 (9 - 10)	78.3%	0.000
Snoring<br>*(which wakes the snorer or affects others)*	8.5 (7.25 - 10)	75.0%	8 (6 - 9)	62.2%	8 (6 - 9)	56.5%	0.023	7 (5 - 8)	38.5%	8 (7 - 9)	71.7%	0.005
Feeling in control of thoughts and feelings	8.5 (8 - 10)	75.0%	8 (6 - 10)	56.1%	7 (6 - 9)	44.7%	0.002	6 (5 - 8)	30.8%	8 (7 - 9)	67.4%	0.000
Confidence to engage in social activity	9 (8 - 10)	75.0%	8 (7 - 10)	67.2%	8 (7 - 10)	63.5%	0.043	8 (6.5 - 9)	53.8%	8 (8 - 10)	71.7%	0.012

Level of Pain	8 (7 - 10)	72.9%	8 (6 - 10)	55.6%	8 (6 - 9)	58.8%	0.091	7 (5 - 8)	33.3%	9 (8 - 10)	80.4%	0.000
Mood Swings	8 (7.75 - 10)	72.9%	8 (6 - 10)	56.7%	7 (5 - 9)	41.2%	0.004	6 (5 - 8)	28.2%	8 (7 - 9)	52.2%	0.001
Self-harm behaviours or thoughts	8.5 (7.75 - 10)	72.9%	8 (5 - 10)	48.9%	9 (7 - 10)	61.2%	0.004	7 (5 - 9)	43.6%	10 (8 - 10)	76.1%	0.000
Self-efficacy *(belief in own ability to succeed)*	9 (7.75 - 10)	72.9%	8 (6 - 10)	57.8%	8 (6 - 10)	48.2%	0.061	6 (5 - 8)	25.6%	9 (8 - 10)	67.4%	0.000
Binge Eating	9 (7 - 10)	70.8%	8 (6 - 10)	60.6%	9 (7 - 10)	64.7%	0.096	7 (6.5 - 9)	46.2%	10 (9.25 - 10)	80.4%	0.000
Cognitive Function *e.g. concentrating, problem-solving, remembering*	8 (7 - 10)	70.8%	8 (7 - 10)	62.8%	8 (6 - 9)	48.2%	0.042	7 (5.5 - 8)	30.8%	8.5 (7 - 9)	63.0%	0.001
Preoccupation with thoughts of food	9.5 (7 - 10)	70.8%	8 (6 - 10)	58.9%	8 (7 - 10)	61.2%	0.103	7 (5 - 8)	38.5%	9 (8 - 10)	80.4%	0.000
Overall quality of life, health and well-being	10 (7 - 10)	70.8%	9 (7 - 10)	64.4%	8 (6 - 10)	54.1%	0.042	7 (6 - 8.5)	38.5%	9 (8 - 10)	67.4%	0.003
Emotional Eating	9.5 (7 - 10)	70.8%	9 (7 - 10)	62.2%	9 (7 - 10)	63.5%	0.224	7 (7 - 8.5)	46.2%	10 (9 - 10)	78.3%	0.000
**Items rated highly important by healthcare practitioners only**												
Medication Use	8 (7 - 10)	68.8%	8 (7 - 10)	67.2%	8 (8 - 10)	78.8%	0.570	8 (7 - 9)	74.4%	9 (8 - 10)	82.6%	0.152
**Items rated highly important by Allied Health practitioners only**												
Physical signs *E.g. hair loss, teeth or gum problems, loss of sensation in hands and feet, skin irritations*	8 (7 - 10)	66.7%	8 (6 - 10)	57.2%	8 (7 - 10)	62.4%	0.251	7 (5 - 9)	38.5%	9 (8 - 10)	82.6%	0.000
Pain interference with day-to-day activities	8 (7 - 10)	64.6%	8 (6 - 10)	51.1%	8 (7 - 9)	67.1%	0.065	7 (6 - 8)	48.7%	9 (8 - 10)	82.6%	0.000
Non-specific symptoms *E.g. headaches, aches & pains, fever, sweats or chills*	8.5 (7 - 10)	66.7%	8 (6 - 9)	52.2%	8 (7 - 10)	60.0%	0.049	7 (6 - 8)	35.9%	9 (8 - 10)	80.4%	0.000
Grazing / Snacking behaviour	9 (6 - 10)	60.4%	8 (7 - 10)	66.1%	9 (7 - 10)	65.9%	0.246	8 (7 - 9)	51.3%	10 (9 - 10)	78.3%	0.000
Feeling guilty or disappointed after eating	9 (7 - 10)	64.6%	8 (6 - 10)	53.9%	8 (7 - 10)	56.5%	0.091	7 (5 - 8)	30.8%	9 (8 - 10)	78.3%	0.000
Thoughts and feelings about physical self	9 (7 - 10)	68.8%	8 (7 - 10)	64.4%	8 (6 - 9)	55.3%	0.077	7 (5 - 8)	28.2%	9 (8 - 10)	78.3%	0.000
Ability to fall asleep	8.5 (7 - 10)	68.8%	8 (6 - 10)	58.9%	8 (7 - 10)	58.8%	0.129	7 (6 - 8)	38.5%	8 (7.75 - 10)	76.1%	0.001
Addictive behaviours *E.g. alcohol, drug use, gambling*	8 (5.75 - 10)	58.3%	8 (5 - 10)	48.3%	8 (7 - 10)	63.5%	0.053	8 (5 - 8)	48.7%	10 (8.25 - 10)	76.1%	0.000
Change in appetite	8 (5 - 10)	54.2%	8 (6 - 10)	56.7%	8 (6 - 10)	61.2%	0.659	7 (6 - 9)	46.2%	8 (7 - 10)	73.9%	0.011
Thoughts and feelings about excess skin or skin folds	8 (7 - 10)	68.8%	8 (7 - 10)	64.4%	8 (6 - 9)	50.6%	0.098	6 (5 - 7.5)	23.1%	9 (8 - 10)	73.9%	0.000
Use of diet pills, laxatives, or intentional vomiting to control weight	8.5 (4.5 - 10)	52.1%	7 (5 - 10)	47.8%	9 (7 - 10)	60.0%	0.002	7 (7 - 9)	43.6%	10 (9 - 10)	73.9%	0.000
Feelings of pleasure from eating	8 (6.75 - 10)	64.6%	8 (6 - 10)	54.4%	8 (7 - 10)	55.3%	0.350	7 (5 - 8)	33.3%	9 (8 - 10)	73.9%	0.000
Avoidance of situations, people or activities because of body image	9.5 (7 - 10)	64.6%	8 (6 - 10)	59.4%	8 (7 - 9.5)	54.1%	0.078	7 (6 - 8)	30.8%	9 (8 - 10)	73.9%	0.000
Amount of food that can be eaten in one sitting	8 (6 - 10)	54.2%	8 (6 - 10)	58.9%	8 (5 - 9.5)	50.6%	0.631	6 (4 - 8)	25.6%	9 (7 - 10)	71.7%	0.000
Satisfaction with sleep	8 (7 - 10)	62.5%	8 (6 - 10)	54.4%	8 (6 - 9)	57.6%	0.332	7 (5 - 8)	41.0%	8 (7 - 9.25)	71.7%	0.018
Experience of stigma or discrimination	8 (6 - 10)	58.3%	7.5 (5 - 10)	48.3%	9 (7 - 10)	57.6%	0.006	7 (6 - 9.5)	41.0%	9 (8 - 10)	71.7%	0.005

** Includes nurses, dieticians, psychologists, and researchers*

Results are presented as median (25th – 75th percentiles) and percentage of participants within each group. Significance indicates differences between groups (a) pre-surgical patients, post-surgical patients and healthcare practitioners, and (b) between medical practitioners and allied health practitioners.
