# Supplementary material for: Development of an item bank and outcome importance survey for the Australian and New Zealand Bariatric Surgery Registry
Source: J Patient Rep Outcomes. 2025 Jul 8;9:85. doi: 10.1186/s41687-025-00918-w (PMC12238453; doi:10.1186/s41687-025-00918-w)
Supplement: Supplementary file 2 — Supplementary Material 2 [file 41687_2025_918_MOESM2_ESM.pdf]

Importance of Patient-Reported Outcomes of Bariatric Surgery

Round One

Thank you for agreeing to take part in this study. This is the first round of a two-part survey.

In this survey we will ask you for some general information and then ask you to rate how important you think it is that we measure a certain outcome of bariatric surgery on a scale of 0 to 10.

This includes outcomes such as general health, physical symptoms, quality of life, mental health and self-image.

Healthcare professionals, and pre- and post-surgical bariatric patients are being asked to complete this survey.

In the second round we will show you how these groups rated each outcome and ask you to vote again, taking into consideration the responses to the first round.

At the end of this survey we will ask you for contact details so we can send you the second round of the survey. These details will be stored separately from your survey responses so that we cannot identify you.

Please answer all of the questions by yourself.

This should take approximately 20 to 30 minutes.

Don't spend too long on the questions; we are looking for your immediate feelings based on your own experience of bariatric surgery.

The information that you provide will remain anonymous.

Please ensure you have read the Patient Information Sheet, outlining what the survey will entail as well as privacy and confidentiality measures, before you take part in this survey.

To allow us to analyse the results of this survey, we need some brief information about you and the type of surgery you have had, or are hoping to undergo. Please choose the most appropriate options.

(Note: this information will not be linked to your name and contact details or be used to identify you)

1. Your sex:

Female ☐ Intersex / Indeterminate ☐

Male ☐ Prefer not to say ☐

2. Your date of birth (month / year): ☐☐ / ☐☐☐☐

3. In which state or territory do you currently reside?

ACT ☐ SA ☐

NSW ☐ TAS ☐

NT ☐ VIC ☐

QLD ☐ WA ☐

4. Your ethnicity (with which ethnic group(s) do you identify?):

(Please select all that apply)

Non-Indigenous Australian ☐ New Zealander ☐

Indigenous Australian or Torres Strait Islander ☐ European ☐

Asian ☐ North American ☐

Indian ☐ South American ☐

Middle Eastern ☐ African ☐

Other ☐ Prefer not to say ☐

Please specify: --

5. Which of the following most accurately describes your employment status?

- Working full-time / Self-employed ☐
- Working part-time / Casual ☐
- Unemployed, seeking work ☐
- Unemployed, not seeking work ☐
- Other ☐
- Student / Apprentice ☐
- Housewife / Househusband ☐
- Unable to work ☐
- Retired ☐

Please specify: ....

6. What is the highest level of education you have completed?

- Less than year 12 or equivalent ☐
- Year 12 or equivalent ☐
- Trade / Technical / Vocational qualification ☐
- Undergraduate degree ☐
- Postgraduate degree ☐

7. Have you already undergone bariatric surgery?

No ☐ (If no, please go to question 13)

Yes ☐ (If yes, please go to question 8)

8. Which weight loss operation did you undergo?

(If you have had more than one operation, please select your first operation type)

- Adjustable gastric band (LAGB) ☐
- Sleeve gastrectomy (LSG) ☐
- Other ☐
- Roux-en-Y gastric bypass (RYGB) ☐
- One anastomosis gastric bypass (OAGB) ☐

Please specify: ....

9. Date of your weight loss surgery (month / year): ☐☐ / ☐☐☐☐

(If you have had more than one operation, please enter the date of your first operation)

10. Have you had any revisional procedures?

No ☐ (If no, please go to question 14) Yes ☐

If yes, please indicate what type:

(if you have had more than one revisional procedure, please select all that apply)

Re-sleeve	☐	RYGB addition of band	☐
Sleeve conversion to RYGB	☐	Port revision	☐
Sleeve conversion to OAGB	☐	Band revision	☐
Sleeve Lavage	☐	Band reversal	☐
Sleeve dilatation	☐	Band conversion to Sleeve	☐
Revision of RYGB	☐	Band conversion to RYGB	☐
Reversal of RYGB	☐	Band conversion to OAGB	☐
RYGB conversion to OAGB	☐	Revision of OAGB	☐
RYGB conversion to Sleeve	☐	Reversal of OAGB	☐
RYGB dilatation	☐	OAGB conversion to RYGB	☐
RYGB division of adhesions	☐	OAGB conversion to Sleeve	☐
Other	☐	OAGB dilatation	☐

Please specify: --

11. Date of your revision procedure (month / year): ☐☐/☐☐☐☐

(If you have had more than one revision procedure, please fill in the text box below)

If you have had more than one revisional procedure please write the type and date of each below:

12. Please indicate the reason(s) you underwent revisional surgery:

13. If you are awaiting surgery, which weight loss surgery option are you hoping to undergo?

(If you have already undergone surgery, skip to question 14)

Adjustable gastric band (LAGB) ☐ Roux-en-Y gastric bypass (RYGB) ☐

Sleeve gastrectomy (LSG) ☐ One anastomosis gastric bypass (OAGB) ☐

Other ☐ Not sure ☐

Please specify: --

14. Do you currently have diabetes?

No ☐ Yes ☐

If yes, please indicate what type:

Type 1 ☐

Type 2 ☐

Gestational ☐

15. Which of the following additional services have you been offered by your surgeon or their team, and which have you used?

(If you are awaiting surgery, do not fill in the 'used post-surgery' column)

	Offered	Used **Pre-Surgery**	Used **Post-Surgery**
Psychologist, psychiatrist or counsellor	☐	☐	☐
Bariatric Physician	☐	☐	☐
GP with interest in bariatrics	☐	☐	☐
Dietician	☐	☐	☐
Exercise Physiologist	☐	☐	☐
Bariatric Nurse Specialist	☐	☐	☐
Endocrinologist	☐	☐	☐
Diabetes Specialist	☐	☐	☐
Hepatologist	☐	☐	☐
Eating Disorder Specialist	☐	☐	☐
Cardiothoracic Physician	☐	☐	☐
Plastic Surgeon	☐	☐	☐
None of the above	☐	☐	☐
Other	☐	☐	☐

Please Specify: --

Thank you for providing that information.

The remaining survey contains questions about various aspects of health and well-being following bariatric surgery. You will be asked to rate how important you think it is that we measure a certain outcome on a scale of 0 to 10.

If you think an outcome has a significant impact on bariatric patients during their surgery and recovery, and should be measured by doctors and researchers, then rank the outcome towards the 'Extremely Important' end of the scale. If you think an outcome does not have an impact on bariatric patients, and should not be measured, rank it towards the 'Not Important' end of the scale.

Some of the outcomes will be accompanied by a definition or example to ensure you understand the outcome you are evaluating.

For example:

Circle how important you think it is that this outcome is measured

Not Important ... Extremely Important

**Perception of Surgery**											
Outcome → Satisfaction with surgery	0	1	2	3	4	(5)	6	7	8	9	10
Outcome → Decision remorse<br>Definition → (feeling of anxiety or regret about the decision to undergo surgery)	0	1	2	3	4	5	6	7	8	9	10

If you are unsure about the outcome, or if you have thoughts or questions, we encourage you to leave a note at the end of the section.

SECTION ONE: Physical Outcomes

The following section lists **physical outcomes** of bariatric surgery.

Please rate **how important you think it is that each outcome of bariatric surgery is measured** to better understand how surgery affects patients.

Please circle the number between 0 and 10 that represents your opinion.

	Not Important										Extremely Important
**General Health**											
Co-morbidities (e.g. diabetes, hypertension, sleep apnoea)	0	1	2	3	4	5	6	7	8	9	10
Medication use	0	1	2	3	4	5	6	7	8	9	10
Physical signs (e.g. hair loss, teeth or gum problems, loss of sensation in hands and feet, skin irritations)	0	1	2	3	4	5	6	7	8	9	10
Non-specific symptoms (e.g. headaches, aches & pains, fever, sweats or chills)	0	1	2	3	4	5	6	7	8	9	10
Digestive symptoms (e.g. strong burping or belching, indigestion, urgent bowel movements, constipation)	0	1	2	3	4	5	6	7	8	9	10
Weight/surgery-specific symptoms (e.g. vomiting, regurgitation, heartburn, nausea, shortness of breath)	0	1	2	3	4	5	6	7	8	9	10
General physical health (e.g. fitness, strength, endurance)	0	1	2	3	4	5	6	7	8	9	10
Mobility (e.g. ability to walk, climb stairs, lift/carry groceries, bend or kneel)	0	1	2	3	4	5	6	7	8	9	10
Ability to care for oneself (e.g. dressing, bathing, grooming, or eating)	0	1	2	3	4	5	6	7	8	9	10
Energy levels / Fatigue	0	1	2	3	4	5	6	7	8	9	10
Level of pain	0	1	2	3	4	5	6	7	8	9	10
Pain interference with day-to-day activities	0	1	2	3	4	5	6	7	8	9	10

	Not Important										Extremely Important
**Eating**											
Ability to eat different types of food	0	1	2	3	4	5	6	7	8	9	10
Amount of food that can be eaten in one sitting	0	1	2	3	4	5	6	7	8	9	10
Ability to have food and drink together	0	1	2	3	4	5	6	7	8	9	10
Tolerance for alcohol	0	1	2	3	4	5	6	7	8	9	10
Change in appetite	0	1	2	3	4	5	6	7	8	9	10
**Sleep**											
Ability to fall asleep	0	1	2	3	4	5	6	7	8	9	10
Quality of sleep	0	1	2	3	4	5	6	7	8	9	10
Satisfaction with sleep	0	1	2	3	4	5	6	7	8	9	10
Snoring (which wakes the snorer or affects others)	0	1	2	3	4	5	6	7	8	9	10
**Sexual Activity**											
Physical ability to engage in sexual activity	0	1	2	3	4	5	6	7	8	9	10
Interest in sexual activity	0	1	2	3	4	5	6	7	8	9	10
Enjoyment / pleasure from sexual activity	0	1	2	3	4	5	6	7	8	9	10
Satisfaction with sex life	0	1	2	3	4	5	6	7	8	9	10

Do you have any comments regarding the above **physical** outcomes? Do you think there are any important physical outcomes that aren’t listed?

SECTION TWO: Quality of Life and Psychosocial Outcomes

The following section lists **quality of life and psychosocial outcomes** of bariatric surgery.

Please rate **how important you think it is that each outcome of bariatric surgery is measured** to better understand how surgery affects patients.

Please circle the number between 0 and 10 that represents your opinion.

	Not Important										Extremely Important
**Perception of Surgery**											
Satisfaction with surgery	0	1	2	3	4	5	6	7	8	9	10
Decision remorse (feeling of anxiety or regret about the decision to undergo surgery)	0	1	2	3	4	5	6	7	8	9	10
**Quality of Life**											
Overall quality of life, health and well-being	0	1	2	3	4	5	6	7	8	9	10
Satisfaction with quality of life	0	1	2	3	4	5	6	7	8	9	10
Normality (Feeling able to live a ‘normal’ life)	0	1	2	3	4	5	6	7	8	9	10
Outlook on life and expectations for the future	0	1	2	3	4	5	6	7	8	9	10
**Social**											
Level of social activity	0	1	2	3	4	5	6	7	8	9	10
Confidence to engage in social activities	0	1	2	3	4	5	6	7	8	9	10
Relationship with spouse/partner or developing intimate relationships	0	1	2	3	4	5	6	7	8	9	10
Relationships with family members	0	1	2	3	4	5	6	7	8	9	10

	Not Important										Extremely Important
Relationships with friends	0	1	2	3	4	5	6	7	8	9	10
Support network (e.g. family, friends, professionals, online/in-person support groups)	0	1	2	3	4	5	6	7	8	9	10
Feelings of social isolation	0	1	2	3	4	5	6	7	8	9	10
Experience of stigma or discrimination	0	1	2	3	4	5	6	7	8	9	10

**Mental Health and Emotional Well-Being**											
Overall mental health	0	1	2	3	4	5	6	7	8	9	10
Mood swings	0	1	2	3	4	5	6	7	8	9	10
Depression	0	1	2	3	4	5	6	7	8	9	10
Anhedonia (loss of pleasure and/or interest in people, things or activities)	0	1	2	3	4	5	6	7	8	9	10
Self-harm behaviours or thoughts	0	1	2	3	4	5	6	7	8	9	10
Suicidal thoughts	0	1	2	3	4	5	6	7	8	9	10
Anxiety	0	1	2	3	4	5	6	7	8	9	10
Fear of negative evaluation (e.g. worry about how others perceive or judge you)	0	1	2	3	4	5	6	7	8	9	10
Feeling in control of thoughts and feelings	0	1	2	3	4	5	6	7	8	9	10
Impulsivity (acting on impulse or without thinking)	0	1	2	3	4	5	6	7	8	9	10
Irritability (feeling easily annoyed or short-tempered)	0	1	2	3	4	5	6	7	8	9	10
Coping (ability to deal with stress or difficulties)	0	1	2	3	4	5	6	7	8	9	10

	Not Important										Extremely Important
Self-Efficacy (belief in own ability to succeed)	0	1	2	3	4	5	6	7	8	9	10
Cognitive function (e.g. concentrating, problem-solving, remembering)	0	1	2	3	4	5	6	7	8	9	10
Addictive behaviours (e.g. alcohol, drug use, gambling)	0	1	2	3	4	5	6	7	8	9	10

**Eating Behaviour and Relationship to Food**											
Binge eating	0	1	2	3	4	5	6	7	8	9	10
Emotional eating	0	1	2	3	4	5	6	7	8	9	10
Grazing / snacking behaviour	0	1	2	3	4	5	6	7	8	9	10
Use of diet pills, laxatives or intentional vomiting to control weight	0	1	2	3	4	5	6	7	8	9	10
Eating patterns (healthy and balanced eating patterns)	0	1	2	3	4	5	6	7	8	9	10
Preoccupation with thoughts of food	0	1	2	3	4	5	6	7	8	9	10
Feeling guilty or disappointed after eating	0	1	2	3	4	5	6	7	8	9	10
Feelings of pleasure from eating	0	1	2	3	4	5	6	7	8	9	10

**Self-Esteem and Body Image**											
Self-esteem / self-confidence	0	1	2	3	4	5	6	7	8	9	10
Thoughts and feelings about physical self	0	1	2	3	4	5	6	7	8	9	10
Preoccupation with thoughts about body size and/or shape	0	1	2	3	4	5	6	7	8	9	10
Thoughts and feelings about excess skin or skin folds	0	1	2	3	4	5	6	7	8	9	10

	Not Important										Extremely Important
Feeling in control of weight and appearance	0	1	2	3	4	5	6	7	8	9	10
Avoidance of situations, people or activities because of body image	0	1	2	3	4	5	6	7	8	9	10

Do you have any comments regarding the above **quality of life and psychosocial outcomes**? Do you think there are any important outcomes that aren't listed?

SECTION THREE: Additional Outcomes and Comments

Are there any outcomes of bariatric surgery you think we've missed?

Please write down anything else related to weight loss surgery you think is important to measure, as well as any other comments or questions you have about this study and the outcomes we've raised. We welcome all your views.
